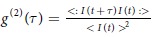# Corrigendum: Localised excitation of a single photon source by a nanowaveguide

**DOI:** 10.1038/srep22823

**Published:** 2016-03-21

**Authors:** Wei Geng, Mathieu Manceau, Nancy Rahbany, Vincent Sallet, Massimo De Vittorio, Luigi Carbone, Quentin Glorieux, Alberto Bramati, Christophe Couteau

Scientific Reports 6: Article number: 1972110.1038/srep19721; published online: 01292016; updated: 03212016

This Article contains typographical errors in Equation 1:


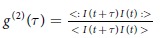


should read: