# Supplementary material for: Localised excitation of a single photon source by a nanowaveguide
Source: Sci Rep. 2016 Jan 29;6:19721. doi: 10.1038/srep19721 (PMC4731774; doi:10.1038/srep19721)
Supplement: Supplementary Information [file srep19721-s1.pdf]

Supporting information

Localised excitation of a single photon source by a nanowaveguide

*Wei Geng[1], Mathieu Manceau[2], Nancy Rahbany[1], Vincent Sallet[3], Massimo De Vittorio[4, 5], Luigi Carbone[5], Quentin Glorieux[2], Alberto Bramati[2], Christophe Couteau[1, 6]**

[1] Laboratory of Nanotechnology, Instrumentation and Optics (LNIO), Charles Delaunay Institute, CNRS UMR 6281, University of Technology of Troyes (UTT), 10000, Troyes, France.

[2] Laboratoire Kastler Brossel, UPMC-Sorbonne Universités, CNRS, ENS-PSL Research University, Collège de France, 4 place Jussieu Case 74, F-75005 Paris, France.

[3] Groupe d'étude de la matière condensée (GEMAC), CNRS, University of Versailles St Quentin, 78035 Versailles Cedex, France.

[4] Istituto Italiano di Tecnologia (IIT), Center for Bio-Molecular Nanotechnologies Via Barsanti sn, 73010 Arnesano (Lecce), Italy.

[5] National Nanotechnology Laboratory (NNL), CNR Istituto Nanoscienze, Via per Arnesano km 5, 73100 Lecce, Italy.

[6] CINTRA CNRS-Thales-NTU UMI 3288, and School of Electrical and Electronic Engineering, Nanyang Technological University, 637553 Singapore.

*Correspondence and requests for materials should be addressed to C.C. (email: christophe.couteau@utt.fr).

μPL of the ZnO nanowire grown by MOCVD method

The micro-PL of a single ZnO NW is spectrally analysed by a high-sensitivity spectrometer (spectrograph: Andor SR500i; f = 500 mm; grating = 300 gr/mm; CCD: Andor DU920P, cooled at –80°C). A strong intrinsic emission of ZnO at 377 nm is observed (Figure S1-a). The asymmetrical shape of the emission spectrum is due to the direct bandgap of ZnO. Meanwhile, a weak surface defect-related emission is also detected from 450 nm to 700 nm (Figure S1-b). The PL intensity ratio between the intrinsic and defect-related emission is about $1/10^4$ which can thus be neglected.

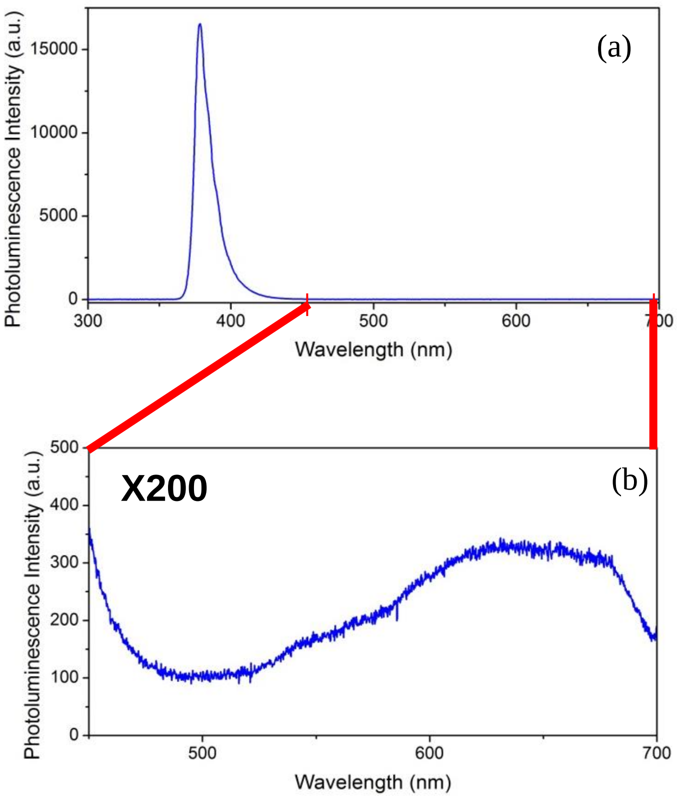

Figure S1. μPL of an individual ZnO NW grown by MOCVD. (a) Strong intrinsic emission of ZnO NW at 377 nm (b) 200× magnified defect emission from 450 nm to 700 nm.

Fabrication and characterisation of the hybrid structure

ZnO nanowires grown by MOCVD were removed from the original sapphire substrate using ultra-sonication in ethanol. Small drops of the suspension of NWs are deposited onto a quartz substrate already coated with a 150 nm layer of PMMA and then dried. In order to totally remove the ethanol on the surface of the NWs, a low power oxygen plasma cleaning is carried then followed by deposition of a layer of 75 nm-thick PMMA containing NCs. The thickness of the PMMA layer was verified using ellipsometry. EBL is carried out under a Raith e-line system. Stripes of 10 μm wide and 1 mm long, as well as numbers for identification were patterned. With a very high electron beam exposure dose (300 $\mu C/cm^2$), the scanned areas of PMMA can be efficiently eliminated down to the first PMMA layer containing no NCs. The purpose of this first layer on the quartz substrate is to make sure that all the NCs are removed from the substrate within the stripes where they are not supposed to be in. After the lithography writing process, the sample is developed in methylisobutylketone/isopropanol (MIBK/IPA) (1:3) solution in order to remove the electron-beam-exposed PMMA. The detail of the configuration and an optical microscopy image are presented in Figure S2. We are not always able to determine whether the NC and the NW are in contact together but they do not have to as long as they are close enough to be optically coupled together.

For the excitation, a 405 nm CW diode laser is focused from the bottom of the sample using a 100X oil-immersion objective (NA = 1.25). The laser spot is slightly defocused to 1.5 μm (measured by burning a hole in the PMMA layer somewhere on the sample) to have a higher coupling efficiency into the NW. A 325 nm CW He-Cd laser is tightly focused from the top with a 40X air objective (NA = 0.6) with a laser spot of 1 μm in diameter (measured by the same way). We used a Plan Achromat objective to collect the signal, which is designed for near-UV to

visible light. We focused the 405 nm laser using a confocal configuration from top. But for the 325 nm laser, we need to use another special UV objective to focus the laser, which has to be from the bottom side. Nevertheless, as the collection set-up is not different for the active or for the passive case, the change of configuration will not affect the results. Indeed, we do need to take care of the coupling of the 325 nm into the NW as we need the nanowire to be excited only by absorption. The intrinsic PL from the NW will then excite the nanocrystal. The magnification of the system is 220X (with a 100X objective and a collection lens of 400 mm). One pixel of the CCD camera from the images stands for 100 nm by 100 nm region on the sample. Considering the observation of the image is under the 300 nm – 400 nm range, the resolution is around 150 nm to 200 nm. The intensity of the signal in figure 6 and figure 4 is around 30 counts/s for the active case, and 50 counts/s for the passive case. This intensity is also related to the excitation laser power. We note that the emission of the PMMA and the substrate under the excitation of 325 nm laser is less than 2 counts/s around 600 nm, thus negligible. It is even less so under the excitation of the 405 nm laser.

(a)

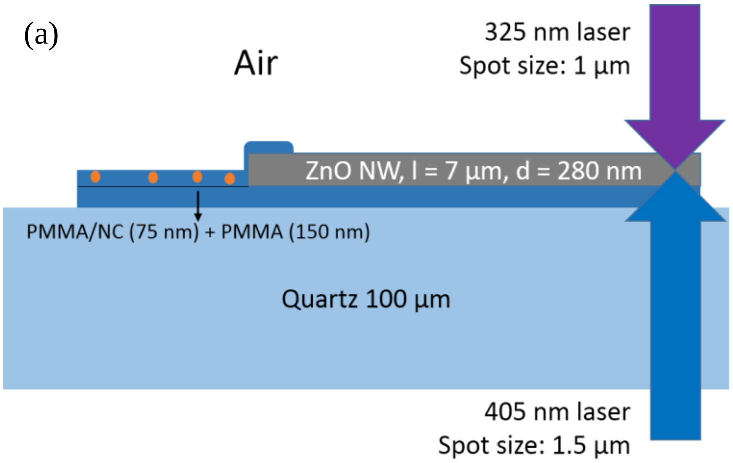

(b)

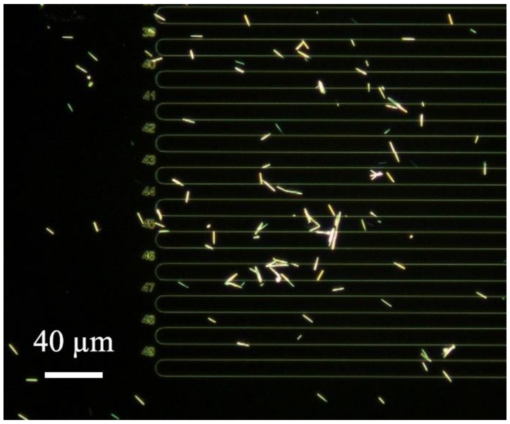

Figure S2. Fabrication of the integration structure. (a) Schematic of the integration structure. (b) Optical microscopy image of the sample in low magnification. The picture shows several single ZnO NWs.

Reproducibility of the integrated structure

The probability of finding an hybrid structure can be controlled by modifying the concentration of NCs and NWs. Here we give another example of the integrated structure discussed in the article. Like the structure presented in Figure 1-b, the excitation of a single CdSe/CdS NC by the ZnO NW presented in Figure S3-a is demonstrated in both passive and active cases in Figure S3-b and -c, respectively. The antibunching behaviour of the single NC has also been verified by giving its coincidence histogram plotted in Figure S3-d. Once again, the fact that $g^{(2)}(0) = 0.15 < 0.5$ proves that the emitter under study in a single photon source. Likewise in the main article, the discrepancy from 0 for the autocorrelation function $g^{(2)}$ is most likely due to spurious light coming from the system and the excitation laser or from the quality of the nanocrystal itself as was shown in Ref. 1.

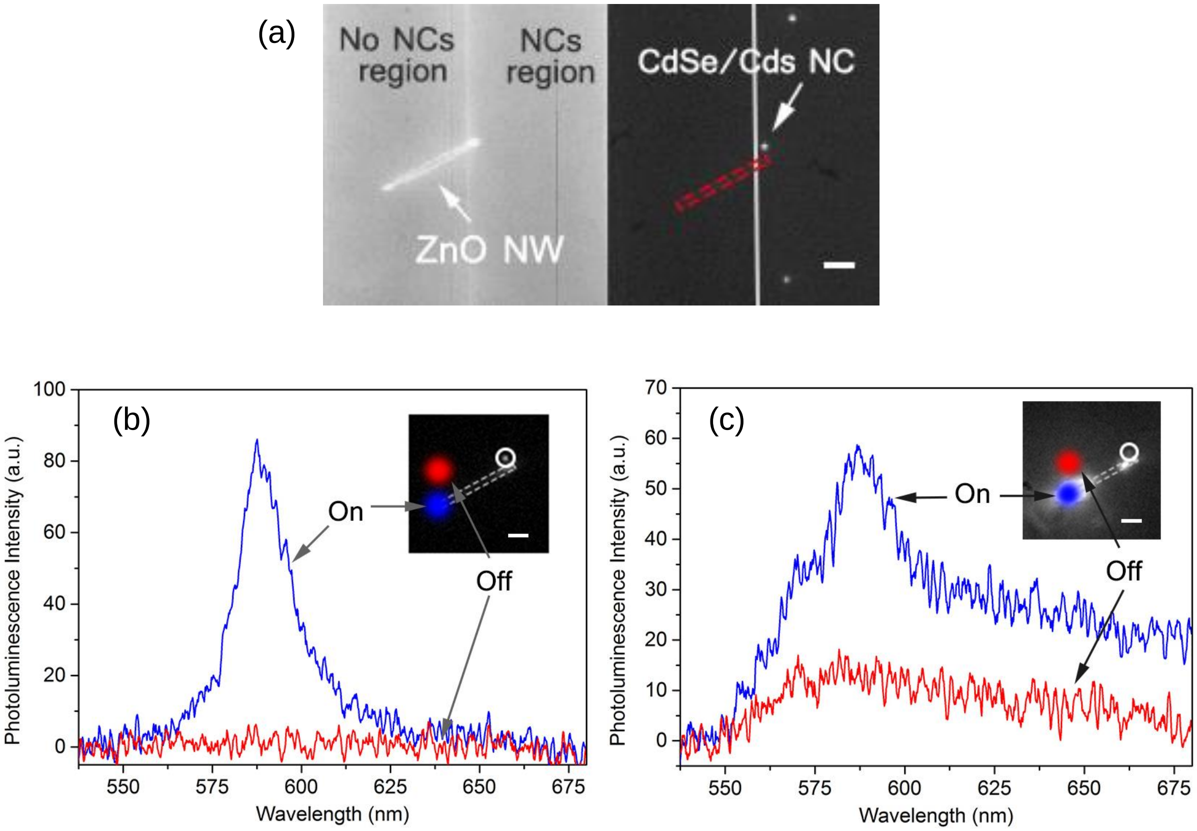

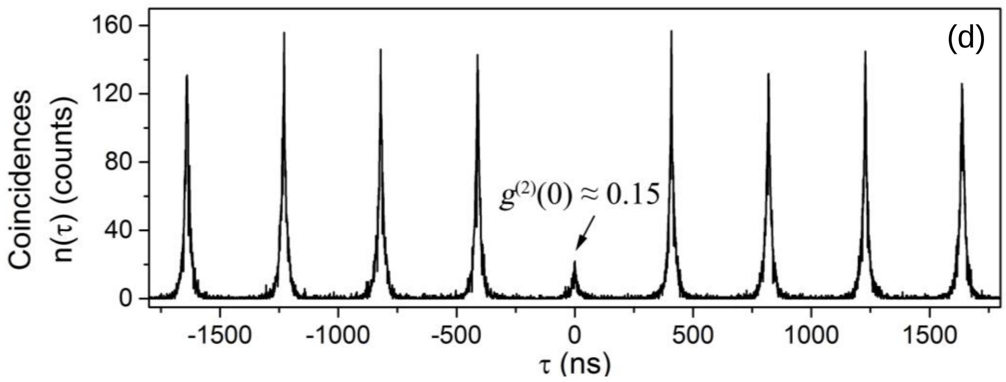

Figure S3. Second example of addressing a single photon source by a nanowaveguide; scale bar is 2 μm. (a) Optical images of the integration structure. (b) and (c) Single CdSe/CdS NC addressed through a passive and an active nanowaveguide, respectively; scale bar in the inset is 2 μm. (d) Coincidence histogram of the CdSe/CdS NC near the ZnO NW.

FDTD simulation of mode propagation in the nanowire waveguide

To understand the passive coupling between the laser beam at 405 nm and the ZnO nanowire waveguide, we use finite-difference time-domain (FDTD) simulation presented in Figure S4 realised by Lumerical. In the simulation, a plane wave with circular polarisation representing the 405 nm laser light is sent to the nanowire from the bottom (right-hand side). Dimensions of the NW were taken to 7 μm long and a diameter of 280 nm to match the experimental conditions. The nanowire cross section is taken as a top view at the centre of the NW. For the ZnO dielectric function inserted in the simulation we used the one from Ref. 2 and the PMMA is considered as a material with index of refraction = 1.482 and we took SiO_2 characteristics from Palik as the material to model the quartz substrate (from Lumerical database). We notice that part of the laser light is totally reflected and confined into the nanowire, propagating to the other end. A propagation beating can be observed which we assign to the fact that the NW is a slight multimode waveguide, as observed in Ref. 3.

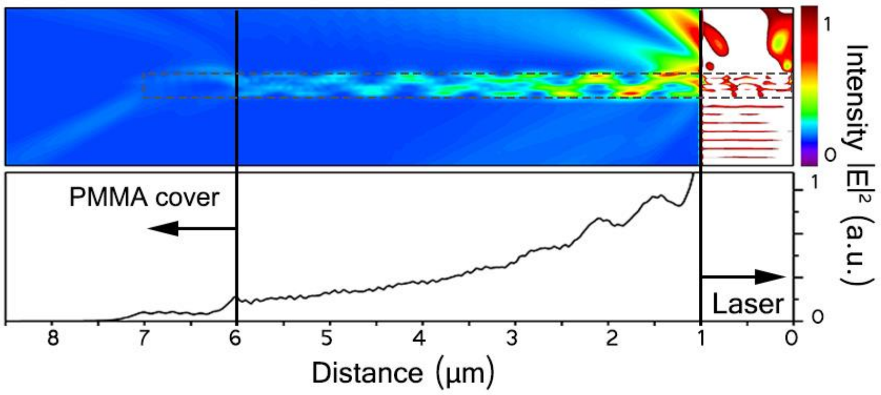

Figure S4. Simulation of a single ZnO NW acting as a passive nanowaveguide: a plane wave at 405 nm is sent on the right-hand side of the nanowire. Upper image: side view of the intensity distribution within the NW. The light is first strongly confined in the NW, and then it escapes from the opposite facet. Lower image: integrated intensity distribution along the NW, which indicates the decay trend of the light within the NW. Due to the sudden change of ambient refractive index where PMMA covers the NW, the light is less confined. But a considerable amount of light can still be found coming out with a certain direction.

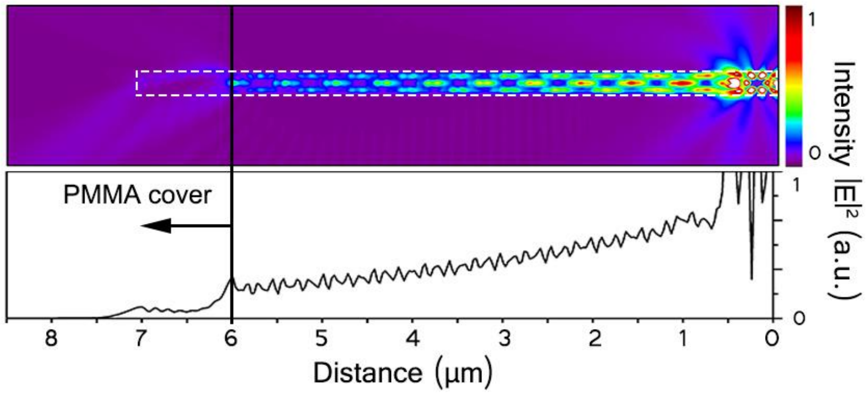

Figure S5. Simulation of a single ZnO nanowire acting as an active nanowaveguide: a dipole is placed on the right end of the nanowaveguide to simulate its own PL. Upper image: side view of the intensity distribution within the nanowaveguide. Lower image: integrated intensity decay along the nanowaveguide axis.

In the active coupling case, using a laser beam at 325 nm and the ZnO NW waveguide, a single dipole is placed on one end of a nanowire to model the intrinsic micro-PL of the material. The wavelength emission of ZnO is included in the material data imported from Ref. 2. Otherwise we use the same properties for the PMMA and the substrate than for the previous passive case. The simulation results, presented in Figure S5, shows that, due to the high refractive index difference as in the passive case (here for 380 nm, $n_{ZnO} = 2.48$, $n_{air} = 1$ and $n_{PMMA} = 1.5$)[2], light is strongly confined along the nanowire. We can observe that the light travels more than 1 μm out of the nanowire which is sufficient to excite nanoemitters nearby the end-facet. Similarly to the passive case, multimode waveguiding is also observed in the simulation curve.

Estimation of the NC excitation addressing efficiency

The excitation addressing efficiencies, respectively in the passive (P_{P}) or active (P_{A}) case, have been defined as the ratios of the photoluminescence intensities of the NC when excited via the nano-waveguide ($I_{\mathrm{WG}}^{\mathrm{P}}$ or $I_{\mathrm{WG}}^{\mathrm{A}}$), over the PL intensities collected under direct NC excitation ($I_{\mathrm{D}}^{\mathrm{P}}$ or $I_{\mathrm{D}}^{\mathrm{A}}$), the latter carried out using standard optical objectives (D for direct).

$$P_{\mathrm{P}} = \frac{I_{\mathrm{WG}}^{\mathrm{P}}}{I_{\mathrm{D}}^{\mathrm{P}}} \text{ and } P_{\mathrm{A}} = \frac{I_{\mathrm{WG}}^{\mathrm{A}}}{I_{\mathrm{D}}^{\mathrm{A}}} \qquad (1A)$$

Two further experimental parameters, which are the coupling efficiencies $\eta_{\mathrm{C}}^{\mathrm{P}}$ or $\eta_{\mathrm{C}}^{\mathrm{A}}$ and the waveguided light propagation efficiencies $\eta_{\mathrm{WG}}^{\mathrm{P}}$ or $\eta_{\mathrm{WG}}^{\mathrm{A}}$ must be considered as well. The coupling efficiencies encompass the fact that for a given incoming laser light power, not all of the light is coupled into the ZnO nano-waveguide. For the passive case, a value of $\eta_{\mathrm{C}}^{\mathrm{P}}$ = 7 % was obtained by direct laser transmission measurement at 405 nm. Whereas, in the active configuration, we measured an absorption of the 325 nm laser of $\eta_{\mathrm{C}}^{\mathrm{A}}$ = 35 % due to the difference between the NW diameter (280 nm) and the Gaussian laser beam spot (around 1 µm) for a given excitation laser power. We also need to take into account the fact that the internal quantum efficiency (IQE) of ZnO is not 100 % for the excitonic emission under 325 nm excitation but rather close to η_{ZnO} = 60 % as shown in Ref. 4.

As for the waveguiding efficiency, it is related to the propagation losses due to the re-absorption by the NW itself and light scattering caused by surface roughness and defects. We cannot presently measure these losses with our setup as there are not observable in the far-field. They could be deduced from FDTD simulations but will be highly dependent on the model used for the refractive index. We note that the proximity of the nanowire does not alter the efficiency

estimation. Indeed, the NC is too far in Figure 1 to be disturbed by the presence of the NW (other than being excited by it obviously). In another situation, it could be the case but one could use a different sample fabrication to avoid such an issue (by placing the layer with emitters above the layer with nanowires for instance).

We experimentally find P_{P} = 0.7 % in the passive case and P_{A} = 1.1 % in the active case. As mentioned, one needs to take into account the coupling efficiencies and the waveguiding efficiency in both cases, thus replacing $I_{\mathrm{D}}^{\mathrm{P}}$ by $I_{\mathrm{D}}^{\mathrm{P}}\eta_{\mathrm{C}}^{\mathrm{P}}\eta_{\mathrm{WG}}^{\mathrm{P}}$ and $I_{\mathrm{D}}^{\mathrm{A}}$ by $I_{\mathrm{D}}^{\mathrm{A}}\eta_{\mathrm{C}}^{\mathrm{A}}\eta_{\mathrm{WG}}^{\mathrm{A}}\eta_{ZnO}$ in order to compensate for the coupling and propagation losses. Without knowing the propagation losses but only using the measured coupling efficiencies, we deduce excitation efficiencies of 10 % for the passive case and 1.89 % for the active case. These two addressing efficiencies are in fact lower limits of our hybrid system as we cannot account for all the losses like scattering and re-absorption processes.

We stress that the above-definition used for the addressing efficiency is in fact set-up dependent and will depend on the optics used for the collection of the light from the nanoemitter. In order to be compared to other systems, one needs to use a figure of merit that can be compared to with another system. With our hybrid system, we can define a β-factor with the usual assumptions so that β = F/(1+F) where F is the Purcell factor given by $\Gamma/n.\Gamma_0$ where Γ_0 is the lifetime in free space of the emitter, n is the index of the medium (here it is PMMA, so n ~ 1.5 at 620 nm) and Γ is the measured lifetime. As a matter of fact, we did measure our emitter's lifetime (not shown) but it is known in these dot-in-rod systems that the lifetime can be quite complex with multiple decay rates due to multiple exciton transitions which depend on the excitation power but also differ from one dot-in-rod to another (Ref. 5) thus rendering this method uncertain in our case.

We could also use the parameter G called the normalised free-space coupling efficiency and defined by:

$$G = \frac{3}{8\pi} . \Omega_{\mu} . \eta^2$$

where $\Omega_{\mu}/(8\pi/3)$ is the normalised (to $8\pi/3$) solid angle fraction covered by the focusing optics (in our case the nanowaveguide or the microscope objective) weighted by the radiation pattern of the emitter (supposed to be a dipole in our case). η is the overlap of the incident radiation with this dipole radiation pattern (for more details, please refer to [6]). We note that $\eta = 1$ for perfect overlap. Nevertheless, this parameter G is valid when one deals with a 'true' emitting dipole which is not our case. It is more or less a 'true' linear emitting dipole but since we are not exciting the emitter in resonance, it is certainly not the case for the absorption behavior of the emitter. Moreover, it would be difficult to estimate the overlap η by FDTD as it does not know how to deal with a source other than a dipole. In this case, we estimated the absolute efficiency of our system in order to estimate the absolute addressing efficiency $\eta_{a.}$ The Table S1 presents all the different losses of our set-up. We have few cases to separate: first of all, we want to differentiate the excitation via microscope objective on one hand and via the waveguide on the other hand. Then in the case of the nanowaveguide excitation, we need to differentiate between the active case and the passive case. For that, Table S1 summarises all the different types of losses for the free-space excitation: common losses to all (amounting to 0.52 10^{-2}) and objective losses (amounting to 1.3 10^{-4}). For the nanowire excitation, we have: common losses to all (amounting to 0.52 10^{-2}) and NW losses (amounting to 1. 10^{-2} for the active case and 0.96 10^{-2} for the passive case). Considering how many counts in total we have on the CCD camera (1.5 10^{4} for the active case only) and considering how much power was put in with the laser (P = 1.6 μW, resulting in the creation of 5.10^{12} photons/s) we can then estimate the

**Common losses (µPL system+NC)**	Name	Value
CCD camera gain (gain of 5)	$g = 1/5$	0.2 (manufacturer)
CCD camera quantum efficiency (at 580 nm)	η_{ccd}	0.83 (manufacturer)
Transmission monochromator	η_{mono}	0.65 (measured)
Transmission µPL system	$\eta_{\mu PL}$	0.33 (measured)
Quantum efficiency nanocrystals	η_{NC}	0.73 (measured)
Collection objective efficiency (NA=1.2)	η_{coll}	0.2 (estimated from specs)
Total common losses	η_{comm}	$0.52\ 10^{-2}$
**Microscope objective excitation**		
Addressing efficiency/Excitation	η_{MO}	$1.3\ 10^{-4}$ (estimated from specs)
**Nanowaveguide excitation**		
ZnO NW absorption (active case)	$\eta_{abs,a}$	0.35 (measured)
ZnO NW absorption (passive case)	$\eta_{abs,p}$	0.07 (FDTD)
ZnO NW quantum efficiency	η_{ZnO}	0.6 (literature)
Attenuation along the NW (active case)	$\eta_{NW,a}$	0.17 (measured)
Attenuation along the NW (passive case)	$\eta_{NW,p}$	0.79 (measured)
Propagation losses/scattering (active case)	$\eta_{scatt,a}$	0.29 (FDTD)
Propagation losses/scattering (passive case)	$\eta_{scatt,p}$	0.35 (FDTD)
Total NW losses (active)	$\eta_{NWlosses,a}$	1.10^{-2}
Total NW losses (passive)	$\eta_{NWlosses,p}$	$0.98\ 10^{-2}$
Addressing efficiency (active case)	η_a	1.10^{-4} (FDTD) / $0.6\ 10^{-4}$ (measured)
**Laser power excitation/photon number (active case)**	N_a	1.6 µW / 5.10^{12} (s^{-1})
**Number of integrated counts on the CCD camera (active case)**	N_{mes}	$1.5\ 10^4$ (s^{-1})

Table S1: Estimated absolute losses for our experimental set-up in the case of direct free-space excitation and for nanowaveguiding excitation.

absolute addressing efficiency $\eta_a = 0.6\ 10^{-4}$ which amounts to only 50 % less than the efficiency using the free-space configuration. Moreover, we ran FDTD simulations where we propagate a plane wave within the nanowire to estimate how much of the incoming light from the NW is still present at a point source place 500 nm away from it (estimated through the optical image from Figure 4) for an absorption cross-section of 100 nm^2 (the same absorption cross-section was used for the objective excitation in Table S1) [7]. The simulated addressing efficiency was found to be $\eta_{a,FDTD} = 1\ 10^{-4}$ thus relatively close the one obtained from our experimental data.

Now it is interesting to see whether that η_a could be increased by engineering another type of NW for instance a single mode NW with a diameter of 100 nm and 70 nm away from the NC, not 500 nm. In this case, we find a value of $\eta_a = 0.25\ 10^{-2}$. We must stress that tailoring the excitation coupled with resonant excitation is key for increasing the quality of indistinguishability of the emitted photon. Ref [8] clearly shows that nanocrystals have resonant absorption peaks in their absorption spectrum, similar to other systems such as epitaxial quantum dots. These first promising results are thus a good omen for much more amelioration of our hybrid system.

Waveguiding in the ZnO nanowires

The optical waveguiding images of passive and active cases are shown in Figure S7-a. The simulation of intensity distribution on the bottom surface of the ZnO nanowire are also shown in Figure S7-b for comparison with the optical images. From optical image of the passive case, some interference pattern can be observed, which coincides with the simulated image. As the leaking light in the active case is too low, we can hardly see similar patterns in the optical image from far-field. However, in both cases, experimental and simulated results show good agreement on the maxima output at the tip of the nanowire.

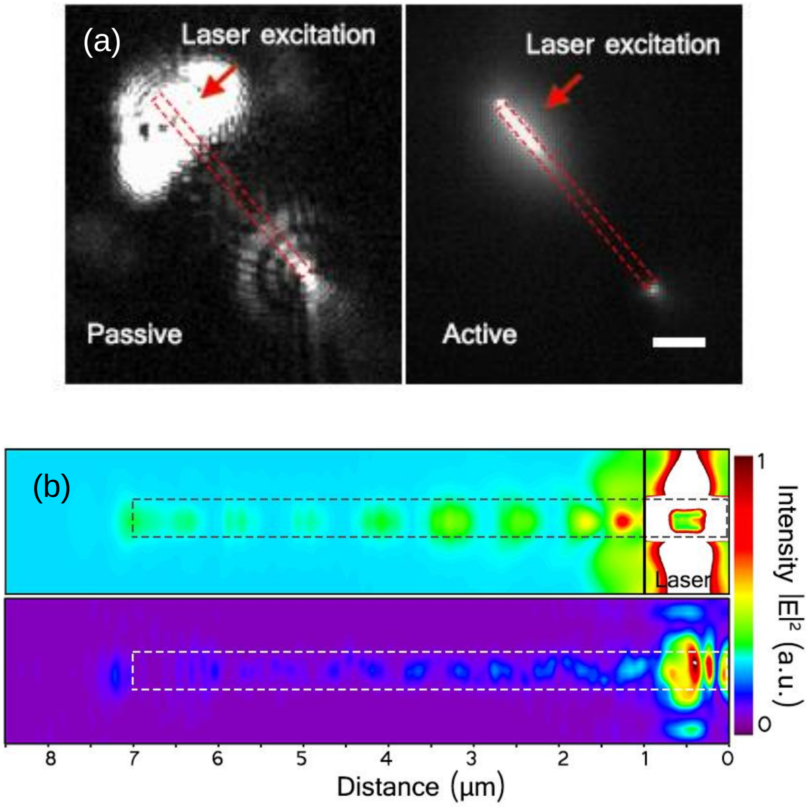

Figure S7. Optical and simulated images of passive and active waveguiding of single ZnO NW. (a) Optical image of the passive (left-hand side) and active (right-hand side) waveguiding; scale bar is 2 µm. (b) Simulated image of intensity distribution on bottom surface of the ZnO NW in passive (upper) and active (lower) waveguiding.

NC emission guided by the nanowaveguide

Instead of indirectly exciting the NC, the nanowaveguides' ability to collect and guide the emission of NCs has also been investigated. To begin with, we analysed this behaviour on nano-waveguides with a NC aggregate made of 2 to 3 nanocrystals (estimated by blinking spectra from the imaging) on their extremity. Figure S8 gives an example of such an integration. By comparing to the far-field collected intensity at the output at the nanowire's end and that of the NCs' emission, we obtain a ratio of 4 - 20% varying from sample to sample. This demonstrates the reversibility of the process which can be useful in future networks made of nanoscale nodes.

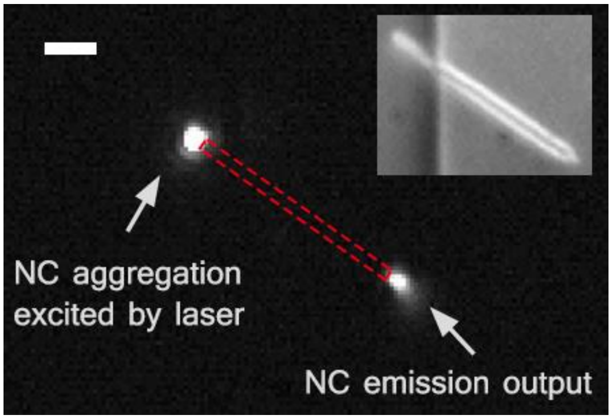

Figure S8. The emission of few NCs excited directly by a laser is guided by the nano-waveguide. Inset is the optical microscopic image of the nanowire with the NCs on one end. Scale bar: 2 µm.

References:

1. Pisanello, F. *et al.* Non-blinking single-photon generation with anisotropic colloidal nanocrystals: towards room-temperature, efficient, colloidal quantum sources. *Adv. Mater.* **25,** 1974–80 (2013).
2. Postava, K. *et al.* Spectroscopic ellipsometry of epitaxial ZnO layer on sapphire substrate. *J. Appl. Phys.* **87,** 7820 (2000).
3. Voss, T. *et al.* High-order waveguide modes in ZnO nanowires. *Nano Lett.* **7,** 3675–80 (2007).
4. Gargas, D. J., Gao, H., Wang, H. & Yang, P. High quantum efficiency of band-edge emission from ZnO nanowires. *Nano Lett.* **11,** 3792–6 (2011).
5. Manceau, M. *et al.* Effect of charging on CdSe/CdS dot-in-rods single-photon emission. *Phys. Rev. B*. **90**, 1–8 (2014).
6. Sondermann, M. *et al.* Maximizing the electric field strength in the foci of high numerical aperture optics. *arXiv*. 0811.2098v3 (2011).
7. H. Htoon *et al.* Light amplification in semiconductor nanocrystals: Quantum rods versus quantum dots. *Appl. Phys. Lett*. 82, 4776 (2003).
8. C. A. Leatherdale *et al.* On the Absorption Cross Section of CdSe Nanocrystal Quantum Dots. *J. Phys. Chem*. B 106, 7619 (2002).
